# The Microbiota–Gut–Brain Axis in Obesity: Inflammation, Appetite Dysregulation, and Feeding Behavior

**DOI:** 10.3390/nu18203409

**Published:** 2026-10-17

**Authors:** Camila Sandoval-Valenzuela, Rodrigo Pacheco

**Affiliations:** 1 Doctorado en Biología Celular y Biomedicina, Facultad de Ciencias, Universidad San Sebastián, Santiago 8580702, Chile; csandovalv11@correo.uss.cl; 2 Centro de Biología Celular y Biomedicina (CEBICEM), Facultad de Ciencias, Universidad San Sebastián, Santiago 8580702, Chile; 3 Laboratory of Neuroimmunology, Centro Científico y Tecnológico de Excelencia Ciencia & Vida, Fundación Ciencia & Vida, Santiago 8580702, Chile; 4 Facultad de Medicina, Universidad San Sebastián, Santiago 8580702, Chile

**Keywords:** gut microbiota, obesity, neuroinflammation, appetite regulation, microbiota–gut–brain axis

## Abstract

Obesity is increasingly recognized as a disorder of bidirectional communication among the gut microbiota, immune system, and neural circuits controlling energy balance and feeding behavior. This review examines the associations and potential mechanistic links between obesity-associated dysbiosis, chronic low-grade inflammation, and impaired appetite regulation through interconnected peripheral and central pathways. We discuss how these signals reach the central nervous system (CNS), and promote microglial and astrocytic activation, impair leptin and insulin signaling, and disrupt hypothalamic POMC and AgRP/NPY neuronal networks. We also examine how microbial metabolites and structural components influence appetite through enteroendocrine hormones, vagal afferents, blood–brain barrier (BBB) integrity, and direct actions on neural and glial cells. Particular emphasis is placed on short-chain fatty acids, tryptophan-derived metabolites, bile acids, and lipopolysaccharide, as well as their effects on glucagon-like peptide-1 (GLP-1), peptide YY (PYY), serotonergic signaling, and mesolimbic reward pathways. Together, these findings support a bidirectional framework in which diet, microbiota function, immune signaling, and neural regulation may reinforce one another during obesity progression. However, the directionality and causal relevance of many of these relationships, particularly in humans, remain incompletely understood. Clarifying these microbiota–gut–brain interactions may identify new targets for restoring appetite control and interrupting obesity progression.

## 1. Introduction

Obesity is a multifactorial disease characterized not only by an imbalance between energy intake and expenditure but also by profound alterations in immune and neuroendocrine pathways [1]. In recent years, the gut microbiota has emerged as a key regulator of host metabolism, contributing to the development of obesity and its associated comorbidities, including insulin resistance, dyslipidemia, and non-alcoholic fatty liver disease (NAFLD) [2].

A hallmark of obesity is chronic low-grade inflammation, which affects multiple tissues and involves activation of inflammatory pathways, including NF-κB, c-Jun N-terminal kinase (JNK), and the inflammasome [3,4]. Experimental studies have demonstrated that this inflammatory response can also occur in the hypothalamus. In rodents, high-fat feeding rapidly activates hypothalamic inflammatory signaling, and experimental inhibition of these pathways can restore hypothalamic insulin signaling and modify food intake, supporting a mechanistic contribution to metabolic dysfunction [5,6,7]. In humans, the evidence is more limited and predominantly associative: neuroimaging studies have identified radiological signatures consistent with mediobasal hypothalamic gliosis in individuals with obesity, while post-mortem studies have reported inflammatory immune-cell infiltration and cellular damage in the hypothalamus [5,6,8,9]. Thus, although preclinical models support a causal contribution of hypothalamic inflammation to metabolic dysfunction, its directionality and functional relevance in human obesity remain less clearly established.

Hypothalamic inflammation may disrupt the function of agouti-related peptide (AgRP) and pro-opiomelanocortin (POMC) neurons, two key first-order neuronal populations involved in the regulation of hunger and satiety [10]. Under physiological conditions, these neuronal populations maintain energy homeostasis through tightly regulated reciprocal signaling within hypothalamic feeding circuits. However, obesity-induced hypothalamic inflammation may disturb this balance, contributing to dysregulated food intake and impaired metabolic control [11,12,13].

At the same time, obesity is associated with significant alterations in gut microbiota composition, characterized by the expansion of microbial populations capable of promoting local and systemic pro-inflammatory responses through structural components and the production of metabolites. These changes are accompanied by the loss of beneficial microorganisms that help maintain metabolic and immunological homeostasis [14,15]. Beyond these compositional alterations, the gut microbiota plays a significant role in regulating host physiology through its effects on nutrient metabolism, gut barrier integrity, and immune system activation [16].

In parallel, growing evidence supports the existence of a bidirectional microbiota–gut–brain axis through which gut-derived signals influence brain physiology and behavior [17]. In particular, the gut microbiota has been shown to modulate the hypothalamic–pituitary–adrenal axis [18], central mechanisms controlling appetite and feeding behavior, and metabolic outcomes through the production of microbial metabolites and the activation of immune signaling pathways [19]. Conversely, alterations in central metabolic regulation may also affect gut microbial composition and function [20].

Taken together, these findings support the concept of obesity as a condition in which gut microbial dysbiosis, chronic inflammation, and hypothalamic dysfunction interact within a self-reinforcing cycle that promotes disease progression. This review explores how obesity-associated alterations in the gut microbiota contribute to inflammatory processes and appetite dysregulation, with a particular focus on the mechanisms linking microbiota-derived signals, hypothalamic inflammation, and metabolic dysfunction.

## 2. Literature Search Strategy

This narrative review was informed by literature searches conducted primarily in PubMed. Searches were performed iteratively using combinations of terms related to obesity, gut microbiota, inflammation, hypothalamic function, appetite regulation, feeding behavior, and microbiota-derived metabolites, including short-chain fatty acids. No predefined publication-date restriction was applied. Studies were initially screened on the basis of title and abstract and were selected according to their relevance to the mechanistic relationships discussed in this review. Particular attention was given to studies addressing microbiota composition and function, peripheral and central inflammatory pathways, gut–brain communication, and appetite-related signaling. When available, primary experimental and human studies were prioritized to support mechanistic or clinical statements, whereas review articles were used primarily to provide broader conceptual context. Because this was a narrative rather than a systematic review, the search was not designed to provide an exhaustive or quantitatively weighted assessment of the available literature.

## 3. Gut Microbiota in Obesity

### 3.1. Alterations in Microbial Composition

Obesity is associated with significant alterations in gut microbiota composition, although findings across studies have not been entirely consistent [21] (Table 1). Early reports suggested an increased Firmicutes-to-Bacteroidetes ratio in individuals with obesity [22]; however, this pattern has not been consistently replicated in human cohorts, highlighting the limitations of high-level taxonomic analyses [23,24].

Despite this variability, several recurring trends have been identified. Obesity is frequently associated with a reduction in beneficial taxa such as *Akkermansia muciniphila* (*A. muciniphila*) [25,26,27] and butyrate-producing bacteria [28], alongside an expansion of opportunistic and pro-inflammatory microorganisms, including members of the Proteobacteria phylum [26,29,30]. In experimental models, some microbiota alterations have been detected before overt metabolic dysfunction [29]; however, temporal precedence alone does not establish a causal role for microbiota composition in obesity.

Recent studies further indicate that obesity-associated dysbiosis varies according to metabolic status and population background. In a Korean cohort, reduced abundances of *Bacteroides*, *Shigella*, *Sutterella*, and *Prevotella* were observed in metabolically healthy obese individuals with normal glucose tolerance, whereas metabolically unhealthy obese subjects exhibited higher levels of these taxa [31]. Similarly, metabolically healthy obesity in other cohorts is generally characterized by a higher abundance of SCFA-producing bacteria, including members of the Lachnospiraceae family and *Faecalibacterium prausnitzii*, whereas insulin-resistant obesity is associated with an enrichment of pro-inflammatory taxa such as *Alistipes* and *Escherichia-Shigella* [32]. These observations suggest that microbial composition may contribute not only to obesity itself but also to the heterogeneity of metabolic outcomes observed among obese individuals.

Supporting a functional contribution of specific microbial strains in experimental models, supplementation with beneficial bacterial strains such as *Lactobacillus bulgaricus*, *Lactobacillus rhamnosus*, and *Bifidobacterium longum* has been shown to attenuate weight gain and reduce the expression of inflammatory markers in brown adipose tissue (BAT) in mice fed a high-fat diet [33].

**Table 1:** Representative microbial taxa associated with metabolic phenotypes and functional outcomes in obesity.

Microbial Feature	Taxonomic Level	Change/Context	Evidence Type and Model	Functional Evidence	References
*Enterobacter cloacae* B29	Strain	Isolated from an individual with obesity	Human-derived isolate colonized into germ-free mice	Colonization promoted adiposity and insulin resistance under the experimental conditions	[34]
*A. muciniphila* GP01 vs GP25	Strain	Different strains show different effects despite belonging to the same species	HFD-fed C57BL/6 mice; strain-specific intervention	GP01 produced stronger improvements in glucose/lipid metabolism, intestinal barrier integrity, endotoxemia and BAT inflammation than GP25	[35]
*A. muciniphila*	Species	Reduced abundance reported in obese mice and humans	Human observational cohorts; HFD-fed mouse interventions	Associated with barrier integrity and metabolic phenotype; supplementation/components improve metabolic outcomes in experimental models	[25,27]
*Lactobacillus bulgaricus, L. rhamnosus* and *Bifidobacterium longum*	Species	Probiotic supplementation during diet-induced obesity	HFD-fed mice; intervention	Reduced weight gain and adipose inflammation and enhanced intestinal IgA-related immune homeostasis	[33]
*L. acidophilus*	Species	Supplementation during diet-induced obesity	HFD-fed mice; intervention	Reduced Gram-negative bacterial abundance and circulating LPS, improved gut barrier integrity and metabolic outcomes	[36]
*Lachnospiraceae* and *Faecalibacterium prausnitzii*	Family + species	Enriched in metabolically healthier obesity phenotype	Human observational study comparing insulin-sensitive vs insulin-resistant obesity	Higher abundance of SCFA-producing taxa was associated with a more favorable metabolic phenotype	[32]
*Alistipes* and *Escherichia–Shigella*	Genera	Enriched in insulin-resistant obesity	Human observational study comparing insulin-sensitive vs insulin-resistant obesity	Enrichment was associated with insulin resistance and a more pro-inflammatory metabolic phenotype	[32]

### 3.2. Functional Shifts in the Obese Microbiome

While taxonomic changes provide valuable insights, functional alterations in the gut microbiome appear to be more consistently associated with obesity. These include changes in microbial metabolic activity, the production of bioactive compounds, and interactions with host immune pathways. The gut microbiota of insulin-resistant obese individuals has been reported to be enriched in pathways related to carbohydrate metabolism, whereas metabolically healthy obese subjects exhibit a greater abundance of pathways involved in amino acid and fatty acid metabolism [32]. These findings suggest that microbial metabolic capacity may contribute to the heterogeneity of metabolic outcomes observed among obese individuals.

In addition to broad metabolic alterations, functional shifts in the gut microbiome may arise from strain-specific host–microbiota interactions. For instance, distinct strains of *A. muciniphila* have been shown to exert differential metabolomic effects in a NAFLD model, with strain GP01 demonstrating greater efficacy in improving hyperlipidemia, hepatic steatosis, and glucose tolerance than strain GP25, despite both strains enhancing intestinal barrier integrity [35]. Notably, only GP01 reduced metabolic endotoxemia, highlighting functional divergence driven by strain-specific genetic and metabolic capacities.

Beyond taxonomic composition, gut microbial communities should be considered as functional ecosystems in which multiple taxa can contribute overlapping metabolic activities. This functional redundancy may partially explain why individuals with markedly different microbial compositions can nevertheless display similar metabolic outputs, whereas subtle strain-level differences may produce distinct host effects. Metagenomic and metaproteomic analyses have shown that taxonomically distinct gut communities can retain overlapping functional repertories, supporting the concept that microbial function cannot always be inferred from taxonomic abundance alone [37]. In obesity, cross-cohort analyses similarly indicate that taxonomic signatures vary among populations, whereas alterations in microbial metabolic pathways may show greater consistency, including changes in lipid biosynthesis and carbohydrate and protein degradation [38]. The response of the microbiome to dietary or pharmacological perturbations is also shaped by ecological resistance and resilience, which determines the magnitude of community disruption and the extent of recovery toward the pre-intervention state [39]. Together with the strain-specific effects described above, these observations suggest that host metabolic outcomes emerge from interactions among microbial metabolic networks, community structure, ecological stability, diet, and host-specific factors rather than from the abundance of individual taxa alone.

#### 3.2.1. Microbial Metabolites

Gut microbiota-derived metabolites play a significant role in host metabolic regulation. Short-chain fatty acids (SCFAs), including acetate, propionate, and butyrate, are key products of microbial fermentation that influence energy homeostasis, immune responses, and gut barrier function [40]. However, the relationship between SCFA concentrations and obesity differs according to the biological compartment analyzed. Human studies have frequently reported higher fecal concentrations of acetate, propionate, and butyrate in individuals with obesity compared with lean controls, although considerable heterogeneity exists across studies [41]. Importantly, fecal SCFA production does not directly reflect microbial production or systemic availability, as it represents the fraction remaining after microbial utilization, colonic absorption, colonocyte metabolism, and intestinal transit [42]. Indeed, human stable-isotope studies have shown that colonically derived SCFAs undergo substantial first-pass utilization, with systemic availability differing markedly among acetate, propionate, and butyrate [43]. Consequently, circulating SCFA concentrations may not parallel fecal concentrations and are additionally influenced by intestinal absorption and hepatic metabolism. Tissue concentrations are even less well characterized, particularly in human obesity, and local SCFA exposure cannot be reliably inferred from either fecal or peripheral blood measurements. Thus, apparently conflicting reports of increased fecal SCFAs but unchanged or altered circulating SCFAs in obesity may reflect differences in production, absorption, utilization, and tissue distribution rather than contradictory biological effects.

SCFAs exert multiple effects on intestinal epithelial function, immune signaling, and host metabolism, which have been extensively discussed in the context of obesity [44]. Experimental studies further indicate that SCFAs can directly modulate inflammatory signaling in peripheral cell types; for example, acetate, propionate, and butyrate suppress TNF-α-induced MCP-1 expression through GPR41/43-dependent modulation of p38 and JNK signaling in human renal cortical epithelial cells [45]. Moreover, propionate has recently been shown to suppress hepatic gluconeogenesis through the activation of AMP-activated protein kinase (AMPK) in experimental models [46].

In addition, microbial metabolism of dietary components generates bioactive molecules such as secondary bile acids and tryptophan-derived metabolites, which can modulate host metabolic pathways and neuroendocrine signaling. These metabolites are increasingly recognized as critical mediators of gut–brain communication.

#### 3.2.2. Structural Components and Microbe-Associated Molecular Patterns (MAMPs)

In addition to metabolites, structural components of bacteria—collectively referred to as microbe-associated molecular patterns (MAMPs)—play a crucial role in host–microbiota interactions. Among these, the implications of lipopolysaccharide (LPS), derived from Gram-negative bacteria, has been extensively studied in obesity-associated inflammation [47].

Disruption of the gut barrier facilitates the translocation of LPS into the systemic circulation, leading to a condition known as metabolic endotoxemia [48]. This process triggers the activation of innate immune pathways and contributes to chronic low-grade inflammation [49]. This topic is discussed in greater detail in Section 4.1.

Several studies have demonstrated that structural and secreted components of *A. muciniphila* can ameliorate diet-induced obesity. Among these, the outer membrane protein Amuc_1100 has been shown to reduce fat mass accumulation, insulin resistance, and dyslipidemia while improving gut barrier integrity in HFD-fed mice through interaction with TLR2 [50]. In addition to membrane-associated factors, the *A. muciniphila* secretome, including extracellular vesicles (AmEVs), has been reported to decrease body weight gain, enhance glucose tolerance, and reduce intestinal permeability in HFD-fed mice [51]. Notably, AmEVs also reduced food intake and alleviated inflammation in adipose tissue and the colon [52]. Furthermore, the secreted protein P9 has been shown to stimulate GLP-1 secretion in both HFD-fed mice and enteroendocrine L cells through interaction with ICAM-1 and induction of IL-6 signaling [53]. Collectively, these findings highlight multiple mechanisms through which *A. muciniphila* exerts beneficial effects on diet-induced obesity.

#### 3.2.3. Diet as an Ecological Driver of Gut Microbiome Function

Diet represents one of the major modifiable ecological drivers of gut microbial composition and function because it determines the substrates available for microbial growth and metabolism. In particular, microbiota-accessible carbohydrates (MACs), including fermentable dietary fibers and resistant starches, provide carbon sources for intestinal microorganisms and support the production of metabolites such as SCFAs. Experimental evidence illustrates the ecological consequences of sustained substrate deprivation: in mice colonized with a human microbiota, consumption of a low-MAC diet progressively reduced microbial diversity, and prolonged exposure across generations resulted in the loss of taxa that could not be restored by dietary MAC reintroduction alone [54]. These findings indicate that dietary composition can influence not only the relative abundance of individual taxa but also ecosystem stability and metabolic capacity.

Human intervention studies further support the capacity of specific fermentable substrates to modify microbiota–host interactions in obesity. In a randomized crossover trial involving adults with overweight or obesity, eight weeks of resistant starch supplementation promoted weight loss and improved insulin resistance while reshaping gut microbiota composition. These effects were associated with enrichment of *Bifidobacterium adolescentis*, altered bile acid metabolism, restoration of intestinal barrier function, and reduced inflammation; complementary experiments in mice supported a functional contribution of *B. adolescentis* to the metabolic effects of resistant starch [55]. Similarly, supplementation with the prebiotic oligofructose in adults with overweight or obesity reduced energy intake and body weight while increasing PYY and decreasing ghrelin concentrations [56]. Together, these findings illustrate how fermentable dietary substrates can influence obesity-related phenotypes through interconnected changes in microbial ecology, metabolite production, intestinal barrier function, and enteroendocrine signaling.

Dietary components other than fermentable carbohydrates can also shape the interactions between microbiota and the immune system. Polyphenols reach the colon in substantial amounts and undergo microbial transformation, while simultaneously influencing microbial community structure. A recent meta-analysis of randomized controlled trials in individuals with overweight or obesity found that polyphenol interventions altered several microbial features and reduced circulating LPS concentrations, although effects on SCFA concentrations were not consistently detected [57]. Fermented foods represent another potential ecological modifier. In a prospective dietary intervention in healthy adults, increased fermented-food consumption increased gut microbial diversity and reduced several inflammatory markers, whereas a high-fiber diet predominantly altered microbial functional capacity, including carbohydrate-active enzymes, with immune responses varying according to baseline microbiota diversity [58]. Although the latter study was not conducted specifically in obesity, it illustrates that different dietary exposures can produce distinct microbiome and immune responses and that baseline community structure may influence individual responsiveness. Thus, dietary effects on the microbiota should not be viewed as uniform: fiber type, resistant starch, polyphenols, fermented foods, habitual diet, and the pre-existing microbial ecosystem collectively determine the metabolic and immunological consequences of dietary exposure.

Taken together, diet- and obesity-associated changes in microbial ecology have consequences that extend beyond local intestinal metabolism. Alterations in microbial metabolites and structural components can modify intestinal barrier integrity, immune tone, enteroendocrine hormone secretion, and vagal signaling, thereby generating peripheral signals with the potential to influence neural pathways involved in energy balance and feeding behavior. SCFAs, LPS, bile acid derivatives, and tryptophan metabolites provide mechanistic links between microbial function and host signaling systems that ultimately converge on appetite-regulating circuits. Thus, changes in gut microbial function may influence brain physiology both indirectly, through immune, endocrine, and neural pathways, and, for selected metabolites, through direct central actions, as discussed below.

### 3.3. From Association to Causality in Obesity-Associated Microbiome Alterations

Experimental models provide some of the strongest evidence that the gut microbiota can contribute functionally to obesity-related phenotypes. Germ-free mice show resistance to diet-induced adiposity and metabolic dysfunction, demonstrating that microbial colonization can influence host energy storage under controlled experimental conditions [59]. Complementarily, transfer of microbiota from human donors with obesity to microbiota-depleted recipient mice has been shown to promote weight gain and inflammatory responses even in the absence of an obesogenic diet, indicating that at least part of the metabolic phenotype can be transmissible through the microbial community [60]. Nevertheless, these models involve substantial manipulation of the host microbial ecosystem and do not establish that obesity-associated dysbiosis precedes or independently causes obesity in humans.

Human evidence is less definitive. Controlled dietary interventions demonstrate that altering substrate availability can reshape the gut microbiota in parallel with changes in metabolic and appetite-related outcomes, as shown for resistant starch and oligofructose supplementation [55,56]. However, these interventions simultaneously modify diet, microbial metabolism, and host physiology, making it difficult to isolate the microbiota as the causal mediator. Cross-cohort and multi-omics analyses can identify microbial functional signatures associated with metabolic phenotype and improve mechanistic resolution beyond taxonomy [25,38], but most human datasets remain observational. Obesity-associated dysbiosis should therefore be considered potentially both a contributor to and a consequence of altered diet, inflammation, and host metabolism, with the relative causal hierarchy likely varying among individuals and disease stages.

## 4. Microbiota-Associated Inflammatory Mechanisms in Obesity

### 4.1. Intestinal Barrier Dysfunction and Endotoxemia

One of the earliest events linking gut microbiota alterations to obesity is the disruption of intestinal barrier integrity [61], leading to obesity-associated intestinal barrier dysfunction, which is characterized by reduced expression of genes encoding junctional proteins, including ZO-1, occludin, E-cadherin and several claudin isoforms [62,63]. These alterations have been validated through tissue immunofluorescence analyses and in vitro functional assays, further supporting their role in promoting increased intestinal permeability.

A major consequence of intestinal barrier disruption is the translocation of microbial components into the bloodstream, a process that contributes to metabolic endotoxemia. Once in the systemic circulation, these molecules promote widespread activation of inflammatory pathways and contribute to chronic low-grade inflammation in multiple tissues.

Among the microbial products implicated in endotoxemia, lipopolysaccharide (LPS), a structural component of the outer membrane of Gram-negative bacteria, has been the most extensively studied. Seminal work by Cani and colleagues demonstrated that chronic elevation of circulating LPS levels is sufficient to induce weight gain, adipose tissue inflammation, and insulin resistance in mice, thereby establishing the concept of metabolic endotoxemia [48,64]. Subsequent studies from the same group further demonstrated that this increase in circulating LPS arises from obesity-associated alterations in gut microbiota composition and intestinal permeability [48].

Mechanistically, circulating LPS activates innate immune signaling pathways, primarily through Toll-like receptor 4 (TLR4), thereby inducing the production of pro-inflammatory cytokines and contributing to insulin resistance and other features of metabolic syndrome. Consequently, several therapeutic strategies have focused on reducing obesity-associated endotoxemia. For example, administration of the phytochemical phlorizin (PHZ) has been shown to increase SCFA production, improve intestinal barrier integrity, and reduce circulating LPS levels, ultimately attenuating insulin resistance in high-fat diet (HFD)-fed mice [65]. Similarly, supplementation with Lactobacillus acidophilus decreases the abundance of Gram-negative bacteria and improves gut barrier function, resulting in lower circulating LPS concentrations and improved metabolic outcomes [36].

The clinical relevance of endotoxemia extends beyond obesity, as elevated circulating endotoxin levels have also been reported in patients with heart failure. This observation highlights the broader pathological consequences of impaired gut barrier function and microbial translocation in chronic inflammatory diseases [66].

Collectively, these findings identify intestinal barrier dysfunction and metabolic endotoxemia as central mechanisms linking obesity-associated gut dysbiosis to chronic systemic inflammation. By facilitating the translocation of microbial products into the circulation, disruption of the gut barrier promotes immune activation in key metabolically active tissues, as discussed below, thereby contributing to the development and progression of metabolic disease.

### 4.2. Adipose Tissue Inflammation

During metabolic endotoxemia, circulating LPS can accumulate in adipose tissue, where it promotes immune-cell recruitment and activation and may favor the polarization of macrophages toward a pro-inflammatory M1-like phenotype. This inflammatory response increases the production of cytokines such as TNF-α and IL-6, which can impair insulin signaling and contribute to insulin resistance [67,68].

Experimental evidence supports a causal role for the gut microbiota in adipose tissue expansion and inflammation. Germ-free mice are protected against diet-induced adiposity and insulin resistance, highlighting the importance of microbial signals in the development of metabolic disease [59]. Similarly, transplantation of gut microbiota from obese individuals is sufficient to induce weight gain and promote adipose tissue inflammation in recipient mice [60].

In addition to structural bacterial components, microbial metabolites also influence adipose tissue homeostasis. Tryptophan-derived metabolites regulate adipocyte function through the miR-181 pathway, thereby affecting energy expenditure, insulin sensitivity, and white adipose tissue inflammation [69]. Likewise, microbiota-derived leucine has been implicated in adipose tissue inflammation through IL-6 trans-signaling, potentially limiting the metabolic benefits of exercise in some individuals [70].

The gut microbiota also shapes the immune landscape of adipose tissue. Microbial depletion reduces regulatory T-cell populations in visceral adipose tissue, whereas microbiota-derived butyrate promotes the expansion of these cells and supports an anti-inflammatory environment [71]. Conversely, obesity-associated microbiota has been linked to increased neutrophil infiltration in visceral adipose tissue, further contributing to local inflammation [72].

Collectively, these findings indicate that the gut microbiota contributes to adipose tissue inflammation through multiple mechanisms, including metabolic endotoxemia, microbiota-derived metabolites, and modulation of immune cells [67,68,69,70,71,72]. These microbiota-driven interactions sustain the chronic low-grade inflammatory state that characterizes obesity and promotes the development of metabolic complications.

### 4.3. Hepatic Inflammation

The gut–liver axis plays a central role in obesity-associated liver disease. Disruption of the intestinal epithelial barrier and gut vascular barrier (GVB) allows microbial products and metabolites to reach the liver through the portal circulation [73], where they can activate immune cells and promote inflammation. This mechanism has been implicated in the development of metabolic dysfunction-associated steatotic liver disease (MASLD), including cases driven by microbial ethanol production by species such as *Klebsiella pneumoniae* [74].

Several microbiota-derived molecules have been identified as mediators of hepatic inflammation. In addition to LPS itself, recent evidence indicates that bacterial extracellular vesicles (bEVs) can cross the intestinal barrier and accumulate in the liver. In diet-induced obese mice, increased portal delivery of LPS-containing bEVs was associated with enhanced hepatic expression of TLR4, macrophage infiltration markers and inflammatory cytokines, suggesting that these vesicles may act as important carriers of pro-inflammatory signals between the gut and the liver [75]. Similarly, gut microbiota-derived D-lactate has emerged as a metabolically relevant microbial metabolite. In obese mice with MAFLD, intestinal trapping of D-lactate reduced hepatic inflammation and fibrosis, supporting a causal role for this microbial metabolite in metabolic and liver dysfunction [76].

Hepatocytes express a wide range of Toll-like receptors (TLRs), enabling them to directly sense microbiota-derived signals that reach the liver through the portal circulation [77]. Among these receptors, TLR9 recognizes unmethylated CpG motifs present in bacterial DNA. Notably, elevated levels of bacterial DNA have been detected in both the blood and feces of patients with MASLD [78,79], suggesting increased hepatic exposure to microbiota-derived nucleic acids. Experimental studies have further demonstrated that TLR9 activation promotes hepatic inflammation and steatohepatitis in vivo [80]. However, despite the growing evidence linking bacterial DNA and TLR9 signaling to liver disease, it remains unclear whether microbiota-derived bacterial DNA directly drives hepatic inflammation during obesity.

In addition to microbe-associated molecular patterns, microbiota-derived metabolites also influence hepatic inflammation and insulin sensitivity. Alterations in microbial tryptophan metabolism have been associated with obesity and liver disease, partly through modulation of the aryl hydrocarbon receptor (AhR). Notably, the tryptophan-derived microbial metabolite 5-hydroxyindoleacetic acid (5-HIAA) improves hepatic insulin signaling and glucose tolerance through AhR-dependent inhibition of mTORC1 signaling in hepatocytes [81]. Additionally, gut microorganisms convert primary bile acids into secondary bile acids, modulating signaling pathways mediated by the farnesoid X receptor (FXR) and Takeda G protein-coupled receptor 5 (TGR5) [82]. These receptors regulate several processes, including inflammatory response during obesity [82,83]. Consequently, alterations in microbiota composition may contribute to obesity-associated changes in bile acid signaling, in addition to classical endotoxemia-mediated mechanisms.

### 4.4. Neuroinflammation and Hypothalamic Dysfunction

Emerging evidence indicates that microbiota-derived signals can influence central neuroimmune homeostasis [84]. In germ-free mice, microglia display an immature phenotype and impaired innate immune responses, whereas recolonization with a complex microbiota partially restores microglial features [85]. SCFA signaling appears to contribute to this process, as FFAR2-deficient mice exhibit microglial alterations resembling those observed under germ-free conditions [85]. Early transport studies in rodents demonstrated that short-chain monocarboxylic acids can cross the blood–brain barrier [86]. In addition, SCFAs can directly affect glial cells in experimental systems; in primary mouse cortical astrocytes, acetate, propionate, and butyrate induce sex-dependent changes in genes related to immune and metabolic signaling [87]. On the other hand, studies in germ-free mice have shown increased BBB permeability and reduced expression of endothelial tight-junction proteins, which can be partially reversed following microbial colonization [88]. Importantly, these studies establish microbiota-dependent regulation of CNS homeostasis in experimental animals but were not performed in models of obesity. Blood–brain barrier dysfunction has also been described in the context of obesity, although the underlying mechanisms and their contribution to hypothalamic inflammation remain incompletely resolved [89].

The inflammatory responses described in the intestine, adipose tissue, and liver may also modify the systemic environment through increased exposure to microbial products, cytokines, lipids, and other inflammatory mediators. These peripheral signals do not necessarily enter the brain directly; rather, they may influence the CNS through endothelial and perivascular signaling at the blood–brain barrier, regions with increased access to circulating signals such as the median eminence, and changes in blood–brain barrier permeability. Experimental obesity models support the involvement of this neurovascular interface. In high-fat diet-fed mice, monocyte-derived macrophages accumulate in the arcuate nucleus, particularly in perivascular and circumventricular regions, and inhibition of macrophage iNOS reduces blood–brain barrier hyperpermeability, inflammatory cytokine production, and reactive astrogliosis [90]. These findings provide a potential mechanistic bridge between the altered systemic inflammatory environment of obesity and hypothalamic neuroinflammation, although direct causal links between inflammation originating in individual peripheral organs and hypothalamic dysfunction remain incompletely established.

In obesity-related experimental models, dietary saturated fatty acids provide a more direct link to hypothalamic inflammation. In mice, saturated fat consumption promotes inflammatory activation of microglia within the mediobasal hypothalamus. Microglial depletion prevents saturated fatty acid-induced hypothalamic inflammation and neuronal stress, demonstrating a mechanistic role for microglia in this response [91]. Human studies, in contrast, primarily provide associative evidence of hypothalamic gliosis and inflammatory alterations in obesity rather than direct evidence that microbiota-derived signals initiate these responses. Experimental evidence from a murine chronic restraint stress model suggests that *A. muciniphila* may attenuate hippocampal neuroinflammation through an SCFA–FFAR2 signaling pathway. In these mice, *A. muciniphila* administration increased fecal and circulating SCFA levels, increased hippocampal FFAR2/GPR43 expression, and reduced inflammatory signaling involving NF-κB, NLRP3, IL-1β, and IL-6. Pharmacological blockade of FFAR2 attenuated the beneficial effects of *A. muciniphila*, supporting the involvement of this receptor in the response [92]. However, whether a similar SCFA–GPR43 pathway operates in obesity-associated hypothalamic inflammation remains unknown. Furthermore, butyrate has been shown to regulate energy balance through primary cilia in hypothalamic AgRP neurons, revealing an additional mechanism by which microbiota-derived metabolites can influence central metabolic control independently of their peripheral actions [93].

Although the mechanisms by which SCFAs act within the hypothalamus remain incompletely understood, recent evidence suggests that GPR43 may contribute to central responses to microbial metabolites. GPR43 expression has been detected in astrocytes and neurons, appearing to be influenced by metabolic status [92,94,95]. Together, these findings support the hypothesis that obesity-associated alterations in gut microbiota composition and metabolite production contribute to hypothalamic inflammation, thereby impairing insulin and leptin signaling and promoting dysregulation of energy homeostasis.

In other pathological contexts, dysbiosis may promote neuroinflammation through multiple complementary mechanisms, including systemic dissemination of pro-inflammatory cytokines, alterations in BBB integrity, recruitment of peripheral immune cells, modulation of vagal signaling, and changes in microbiota-derived metabolites such as tryptophan derivatives [96]. However, these mechanisms have not yet been studied in models of obesity. Figure 1 summarizes the above-mentioned mechanisms by which diet-induced gut dysbiosis promotes inflammation in both peripheral organs and the CNS.

## 5. Microbiota-Mediated Regulation of Appetite

### 5.1. Enteroendocrine Signaling

The gut microbiota plays a critical role in regulating the secretion of hormones involved in appetite control, including GLP-1 and PYY, two key satiety-related gut hormones [97]. These hormones are secreted by enteroendocrine L-cells in response to nutrient and microbiota-derived metabolites and act on both the CNS and peripheral tissues to promote satiety and reduce food intake [98]. Experimental studies in rodents indicate that inulin-type fructans can enhance enteroendocrine satiety signaling [99]. Human intervention studies provide more selective evidence. In a randomized, double-blind, placebo-controlled trial in adults with overweight or obesity, 12 weeks of oligofructose supplementation increased the PYY response, reduced ghrelin concentrations and self-reported energy intake, but did not significantly alter circulating active GLP-1 [56]. Furthermore, in humans, circulating fasting ghrelin levels were positively correlated with the abundance of *Bacteroides*, *Bifidobacterium longum*, and *Parabacteroides distasonis*, whereas negative correlations were observed with *Prevotella* and Prevotellaceae [100], suggesting that gut microbial composition contributes to the regulation of hunger signaling. In addition, a growing body of evidence indicates that microbial metabolites, particularly short-chain fatty acids (SCFAs), are key regulators of enteroendocrine function. Mechanistic evidence from primary colonic cultures and Ffar2-deficient mice indicates that SCFAs stimulate GLP-1 secretion partly through FFAR2/GPR43 signaling [101], providing a direct mechanistic link between microbial fermentation products and satiety signaling.

Beyond peripheral hormone secretion, the gut microbiota also modulates central pathways involved in appetite control by influencing leptin sensitivity. Lower gut bacterial richness has been associated with higher circulating leptin concentrations in both obese and non-obese individuals, suggesting a potential association between gut microbial diversity and leptin regulation [102]. Furthermore, leptin administration suppresses the hypothalamic expression of the orexigenic neuropeptides neuropeptide Y (NPY) and agouti-related peptide (AgRP) in germ-free mice, but not in conventionally colonized animals, highlighting a critical role for the gut microbiota in mediating leptin signaling [103]. Likewise, microbiota depletion or dysbiosis has been associated with reduced expression of anorexigenic neuropeptides, including proglucagon (Gcg) and brain-derived neurotrophic factor (Bdnf), suggesting that microbial signals extend beyond the regulation of enteroendocrine hormone secretion to directly influence hypothalamic and cortical circuits involved in energy homeostasis [103]. Collectively, these findings support the concept that the gut microbiota regulates appetite by integrating enteroendocrine hormone secretion with central neural circuits governing satiety and energy balance.

### 5.2. Vagal Sensing

In addition to endocrine communication, microbiota-derived signals can influence appetite by activating the vagus nerve, a major neural pathway connecting the gastrointestinal tract to the brain. Vagal afferent neurons continuously monitor mechanical, nutritional and hormonal signals originating in the gut and relay this information to the nucleus tractus solitarius (NTS) in the brainstem. These signals are subsequently integrated and transmitted to the paraventricular (PVN) and arcuate nucleus (ARC) of the hypothalamus, two key regions involved in appetite regulation [98,104,105].

Rather than directly sampling the intestinal lumen, vagal afferents rely on specialized intestinal sensory cells that translate luminal stimuli into neural signals. One of the primary mechanisms by which the gut microbiota modulates vagal activity involves enteroendocrine cells. As discussed above, SCFAs stimulate enteroendocrine L-cells to release the satiety hormones GLP-1 and PYY, which activate vagal afferent terminals and convey satiety signals to the brainstem [106]. Likewise, gastrointestinal mediators such as cholecystokinin (CCK) and serotonin (5-HT), released following nutrient ingestion, further promote vagal activation and contribute to the regulation of feeding behavior [104]. Beyond hormone secretion, recent evidence indicates that enteroendocrine cells can form direct glutamatergic synapses with vagal afferent neurons, thereby enabling rapid gut-to-brain communication in response to luminal nutrients [107].

In addition to producing microbial metabolites, several gut microorganisms can synthesize neurotransmitters, including gamma-aminobutyric acid (GABA), dopamine, and acetylcholine. Although these molecules generally do not cross the intestinal epithelium or the blood–brain barrier, they can modulate intestinal epithelial cells, enteroendocrine cells and enteric neurons, thereby indirectly influencing vagal afferent activity [104]. Experimental studies in non-appetite contexts indicate that SCFAs can engage vagal pathways involving GPR41 and/or GPR43 [108,109], supporting the biological plausibility of SCFA-dependent vagal signaling. However, the extent to which this mechanism contributes specifically to appetite regulation remains unclear, despite the well-established role of SCFAs in appetite regulation [110].

Recent evidence has identified a dedicated gut–brain sensory circuit in which microbial flagellin activates TLR5-expressing colonic neuropod cells, thereby inducing PYY release onto vagal afferent neurons and ultimately suppressing food intake. This process, termed neurobiotic sensing, demonstrates that the host can rapidly detect microbial signals through specialized neuroepithelial cells, independently of systemic metabolic responses [111].

### 5.3. Direct Central Actions of Microbial Metabolites

Beyond endocrine and neural communication pathways, the composition of the gut microbiota and its metabolites may directly influence CNS functions involved in appetite regulation [112]. SCFAs exert many of their effects through specific receptors, primarily GPR41 and GPR43, which are expressed in intestinal and immune cells [113] and have recently been reported in several brain populations [92,95,114,115]. In this context, acetate can cross the BBB and reach the CNS. Once in the brain, acetate has been shown to reduce food intake by promoting hypothalamic neuronal activation and altering neuroglial metabolism, including enhanced glutamate–glutamine and GABA cycling, as well as increased lactate production in the hypothalamus [116]. Similarly, intracerebroventricular administration of butyrate has been shown to reduce the expression of neuropeptide Y (NPY) and AgRP, inhibit the excitability of AgRP neurons, and ultimately decrease food intake [93]. Collectively, these preclinical studies demonstrate that experimentally administered SCFAs can exert direct central effects on appetite-regulating pathways in rodents. Acetate provides particularly strong evidence because isotope-tracing studies have shown that colon-derived acetate can reach the brain and accumulate in the hypothalamus [116]. However, whether endogenous microbiota-derived SCFAs reach the human CNS at concentrations sufficient to reproduce these effects remains unresolved.

Emerging evidence has identified GPR43 expression in cortical neurons and astrocytes, with receptor abundance appearing to vary according to the host’s metabolic status [94,95,117]. In addition, da Silva and colleagues demonstrated that GPR43 is expressed in hypothalamic pro-opiomelanocortin (POMC)-expressing neurons and that its expression is reduced in mice fed a high-fat diet [117]. These findings raise the possibility that microbiota-derived SCFAs directly modulate neuronal and glial pathways involved in energy balance and feeding behavior. However, the roles of GPR43 and other SCFA receptors within the CNS remain largely unexplored.

As discussed above, gut microbiota-derived GABA may modulate appetite through mechanisms involving the microbiota–gut–brain axis. In rabbits, increased microbial GABA production was associated with higher food intake and reduced secretion of the anorexigenic hormones CCK, GLP-1, and PYY [118]. However, the underlying mechanisms remain to be identified. Figure 2 illustrates the pathways linking microbiota-derived metabolites to hypothalamic circuits regulating appetite.

Another important microbiota-dependent mechanism involves tryptophan metabolism and activation of the aryl hydrocarbon receptor (AhR). Gut microorganisms metabolize dietary tryptophan into a broad range of bioactive compounds capable of activating AhR signaling in both peripheral and central tissues. Emerging evidence suggests that alterations in microbiota-dependent tryptophan metabolism may influence body weight regulation and overall metabolic homeostasis [119].

Importantly, microbiota-dependent regulation of appetite and microbiota-associated inflammation should not be considered independent pathways. Increased exposure to microbial products such as LPS can promote systemic inflammatory signaling and metabolic endotoxemia in experimental obesity [48,64], whereas SCFAs can simultaneously influence enteroendocrine signaling; in particular, acetate, propionate, and butyrate stimulate GLP-1 secretion through FFAR2/GPR43-dependent mechanisms [101]. These peripheral signals may therefore modify the inflammatory and hormonal environment to which appetite-regulating neural circuits are exposed. Within the hypothalamus, obesity-associated inflammatory responses involve activation of microglia and astrocytes and are linked to impaired leptin and insulin signaling [6,120]. Experimental studies further show that microglial activation can alter hypothalamic neuronal function during saturated-fat exposure [91], while inflammatory disruption of leptin and insulin signaling can modify the responsiveness of POMC and AgRP/NPY neurons involved in food-intake regulation [120,121,122]. Thus, microbiota-derived signals may influence feeding behavior not only through direct endocrine, vagal, or central pathways, but also indirectly by shaping peripheral and central inflammatory responses that converge on neural circuits controlling energy intake.

## 6. Crosstalk Between Inflammation and Appetite: A Systems-Level Feedback Loop

### 6.1. Inflammation-Induced Disruption of Appetite Regulation

Chronic low-grade inflammation is now recognized as a major mechanism underlying obesity-associated dysregulation of appetite. As discussed previously, elevated circulating levels of LPS, pro-inflammatory cytokines, and saturated fatty acids activate inflammatory pathways in the hypothalamus, promoting microglial and astrocytic activation and inducing the local production of TNF-α, IL-1β, and IL-6 [6,120]. These inflammatory signals impair leptin and insulin receptor signaling through activation of the SOCS3, NF-κB, and JNK pathways, resulting in central leptin and insulin resistance [120]. Consequently, anorexigenic POMC neurons become less responsive to peripheral satiety signals, whereas orexigenic AgRP/NPY neurons remain active, ultimately promoting hyperphagia and a positive energy balance [121,122]. Thus, obesity-associated neuroinflammation shifts physiological appetite regulation toward a pathological state characterized by persistent hunger despite elevated levels of anorexigenic hormones.

### 6.2. Diet, Microbiota and Reinforcement of Inflammatory States

The inflammatory alterations associated with obesity are continuously reinforced by dietary habits. Long-term consumption of Western-style diets rich in saturated fatty acids and refined carbohydrates profoundly alters the composition of the gut microbiota, reducing beneficial SCFA-producing bacteria while increasing the abundance of Gram-negative bacteria capable of releasing LPS [48,64]. These changes reduce the secretion of SCFA-dependent satiety hormones, such as GLP-1 and PYY, while promoting a systemic pro-inflammatory state.

At the same time, disruption of intestinal barrier integrity facilitates the translocation of microbial products into the circulation, resulting in metabolic endotoxemia and chronic activation of immune pathways [64]. In parallel, reduced SCFA production limits their anti-inflammatory effects on immune cells and decreases signaling through GPR41 and GPR43, further amplifying inflammatory responses [123]. From a systems-level perspective, these interactions are better understood as interconnected feedback loops rather than as a unidirectional sequence of events. Dietary exposure can modify microbial community structure and function, thereby altering microbial products, intestinal barrier integrity, immune activation, and metabolic signaling [48,64]. These host responses can in turn influence food intake, nutrient availability, and intestinal physiology, creating additional selective pressures on the microbial ecosystem and reinforcing bidirectional host–microbiome interactions [120,121,122,123]. Importantly, gut microbial communities can exhibit resistance and resilience to dietary and other environmental perturbations, and the magnitude and duration of these responses vary substantially among individuals [39]. Ecological analyses of the human gut microbiota have also identified bacterial groups displaying alternative stable abundance states and reduced stability at intermediate abundances, providing empirical support for the concept of microbial “tipping elements” [124]. Accordingly, sustained obesogenic pressures could theoretically shift host–microbiome interactions toward alternative configurations characterized by persistent metabolic and inflammatory dysfunction. However, whether discrete tipping points linking microbiome alterations, neuroinflammation, and appetite dysregulation occur during the development of human obesity remains unknown and will require longitudinal and integrated multi-omics studies.

### 6.3. Feeding Behavior and Hedonic Eating

Beyond homeostatic regulation, chronic inflammation may also affect neural circuits involved in food reward by altering dopaminergic neurotransmission within the mesolimbic reward system, potentially contributing to dysregulated food-reward processing [125,126]. Experimental studies further suggest that microbiota-derived metabolites, vagal signaling, and neuroimmune interactions modulate these circuits, thereby influencing preferences for highly palatable, energy-dense foods [127]. These foods may promote inflammation not only by altering the gut microbiota, but also through their high content of saturated fatty acids, which can activate pro-inflammatory signaling pathways and perturb cellular lipid homeostasis, including TLR-associated signaling, lipid raft organization, and oxidative stress [128,129].

Another microbiota-dependent pathway involves serotonin (5-hydroxytryptamine, 5-HT). Importantly, peripheral and central serotonin constitute largely distinct pools. Peripheral 5-HT is predominantly synthesized by TPH1-expressing enterochromaffin cells in the gastrointestinal tract, whereas central 5-HT is primarily produced by TPH2-expressing serotonergic neurons. Because serotonin does not readily cross the blood–brain barrier, changes in circulating gut-derived 5-HT should not be interpreted as equivalent to alterations in central serotonergic neurotransmission [130].

Gut microorganisms can regulate peripheral serotonin biosynthesis. In mice, Yano et al. demonstrated that indigenous spore-forming bacteria and their metabolites stimulate 5-HT production by colonic enterochromaffin cells, increasing mucosal, luminal and circulating serotonin and modifying gastrointestinal motility and platelet function [131]. These findings establish microbiota-dependent regulation of peripheral 5-HT but do not demonstrate a direct effect on brain serotonin, mood or emotional eating. Microbiota-dependent modulation of tryptophan metabolism and other gut–brain signaling pathways may provide indirect routes through which the intestinal environment influences central serotonergic function; however, direct causal evidence linking microbiota-induced changes in peripheral 5-HT to emotional eating or obesity in humans remains limited.

Figure 3 illustrates a reinforcing loop in which diet-induced gut dysbiosis promotes inflammation, and this state alters neurological pathways involved in feeding behavior.

## 7. Therapeutic Implications and Future Perspectives

The growing involvement of the gut microbiota in obesity-associated metabolic and neuroimmune alterations has stimulated interest in microbiota-targeted interventions. Dietary modulation represents one of the most accessible strategies because fermentable substrates can modify microbial metabolism and the production of bioactive metabolites. In humans with overweight or obesity, supplementation with oligofructose has been associated with modest reduction in body weight and energy intake, together with increased PYY and reduced ghrelin concentrations [56]. Preclinical studies further indicate that supplementation with selected *Lactobacillus* and *Bifidobacterium* strains can attenuate weight gain, improve intestinal barrier integrity, and reduce inflammatory responses during diet-induced obesity [33,36]. Nevertheless, clinical responses to prebiotics and probiotics are heterogeneous, and changes in microbiota composition do not consistently translate into sustained weight loss or metabolic improvement. Recent evidence from randomized clinical trials therefore supports viewing these approaches as complementary rather than stand-alone treatments for obesity [132].

Next-generation probiotics and microbiota-derived peptides may provide more targeted approaches than conventional probiotic formulations. *A. muciniphila* is among the most extensively investigated candidates. Preclinical studies have identified several potentially beneficial mechanisms involving the bacterium itself and its derived components, including the outer membrane protein Amuc_1100, extracellular vesicles, and the secreted protein P9, which influence intestinal barrier integrity, inflammatory signaling, glucose metabolism, and GLP-1 secretion [50,51,52,53]. Importantly, these findings are beginning to translate into human studies. An initial randomized proof-of-concept trial shows that supplementation with live or pasteurized *A. muciniphila* was safe and well tolerated in insulin-resistant individuals with overweight or obesity, with pasteurized bacteria improving several metabolic parameters [133]. More recently, a randomized controlled trial in adults with overweight or obesity who had achieved diet-induced weight loss showed that pasteurized *A. muciniphila* reduced subsequent weight regain compared with placebo [134]. These findings support the translational potential of next-generation probiotics, although larger and longer studies are required to establish efficacy, optimal formulations, responder profiles, and long-term safety.

Interactions between microbial signaling and established anti-obesity therapies represent another emerging area of interest. Microbial metabolites such as SCFAs stimulate GLP-1 secretion through GPR43-dependent mechanisms in experimental systems [101], while *A. muciniphila*-derived P9 can also promote GLP-1 secretion [53]. Conversely, GLP-1 receptor agonists may alter gut microbial composition, raising the possibility of bidirectional interactions between pharmacological treatment and the microbiome. In obese mice, semaglutide-induced metabolic and anorexigenic responses have been accompanied by changes in the gut microbiota composition and microbiota–gut–brain signaling [117]; however, current human evidence remains heterogeneous, and it is not yet clear whether microbiome changes contribute to therapeutic efficacy or occur secondarily to weight loss, reduced food intake, or dietary changes [135].

Direct manipulation of microbial metabolite exposure represents an additional therapeutic strategy. Human intervention studies have begun to test whether increasing SCFA availability can reproduce some of the metabolic effects observed in experimental models. In men with overweight or obesity, acute colonic administration of physiologically relevant SCFA mixtures increased fat oxidation and circulating PYY concentrations, while acetate- and propionate-enriched mixtures also increased resting energy expenditure [136]. Targeted colonic delivery of propionate using an inulin-propionate ester has further been associated with reduced energy intake, weight gain, and abdominal adiposity in adults with overweight [137]. However, results are not uniformly beneficial. In a pilot study, four weeks of oral sodium butyrate improved hepatic and peripheral insulin sensitivity in lean men but not in men with metabolic syndrome, suggesting that host metabolic status and SCFA handling may strongly influence treatment response [138]. Thus, although SCFA-based interventions are mechanistically attractive, optimal delivery route, dose, target population, and long-term efficacy remain unresolved.

Interindividual variation in baseline microbiota composition may also contribute to heterogeneous responses to dietary or microbial interventions, providing a rationale for precision nutrition. Microbiota-based models have shown potential for predicting individual responses to different weight-loss diets [139]; nevertheless, randomized trials of multi-omics-guided personalized diets have not consistently demonstrated superior weight loss compared with high-quality general dietary recommendations [140]. Thus, microbiota profiling is not yet sufficiently validated to guide routine obesity treatment. Rather than attempting to restore a single “healthy microbiota”, future strategies will likely need to integrate microbiome modulation with dietary, behavioral, and pharmacological interventions while accounting for individual metabolic and microbial characteristics.

## 8. Limitations

Several limitations should be considered when interpreting the evidence discussed in this review. First, a substantial proportion of the mechanistic evidence linking the gut microbiota to inflammation, neuroimmune signaling, and appetite regulation derives from experimental animal models, germ-free systems, or in vitro studies, and these findings cannot necessarily be extrapolated directly to human obesity. Human studies remain predominantly observational and frequently cross-sectional, limiting inference regarding the directionality and causal hierarchy among diet, microbiota alterations, inflammation, metabolic dysfunction, and feeding behavior.

Second, substantial methodological heterogeneity exists across microbiome studies, including differences in study populations, dietary exposure, medication use, sequencing approaches, taxonomic resolution, and analytical pipelines. In addition, microbial functions may vary substantially among strains and may be partially preserved through functional redundancy across taxonomically distinct communities.

Finally, measurements of microbiota-derived metabolites are highly dependent on the biological compartment analyzed. As illustrated by SCFAs, fecal concentrations do not necessarily reflect microbial production, systemic exposure, or tissue availability. These limitations highlight the need for longitudinal human studies, controlled interventions, strain-resolved and functional microbiome analyses, and integrated multi-omics approaches to clarify the causal mechanisms underlying microbiota–gut–brain interactions in obesity.

## 9. Conclusions

Obesity is associated with coordinated changes in dietary exposure, gut microbiota composition and function, immune signaling, and neural pathways controlling appetite and energy balance. Experimental models provide mechanistic evidence that specific microbial components and metabolites can alter intestinal barrier function, peripheral inflammation, neuroimmune signaling, and appetite-related pathways. However, human evidence remains predominantly associative, and the directionality and causal hierarchy among diet, microbiota alterations, metabolic dysfunction, inflammation, and feeding behavior remain incompletely resolved.

These limitations are particularly relevant for microbiota-derived metabolites such as SCFAs, tryptophan derivatives, and bile acids, whose concentrations, tissue distribution, receptor-dependent effects, and physiological relevance may differ substantially across experimental models and biological compartments. Similarly, mechanisms established in germ-free animals, genetically modified mice, or cellular systems cannot necessarily be extrapolated directly to human obesity.

The microbiota–gut–brain axis should therefore be considered a dynamic and bidirectional network rather than a single linear causal pathway. Microbiota-targeted nutritional and pharmacological strategies represent promising complementary approaches, but their clinical efficacy, durability, and interindividual variability remain to be established. Longitudinal human studies, controlled interventions, and integrated multi-omics approaches will be essential to distinguish causal mechanisms from adaptive or secondary microbial changes and to identify which components of this network constitute actionable therapeutic targets.

## Figures and Tables

**Figure 1:**
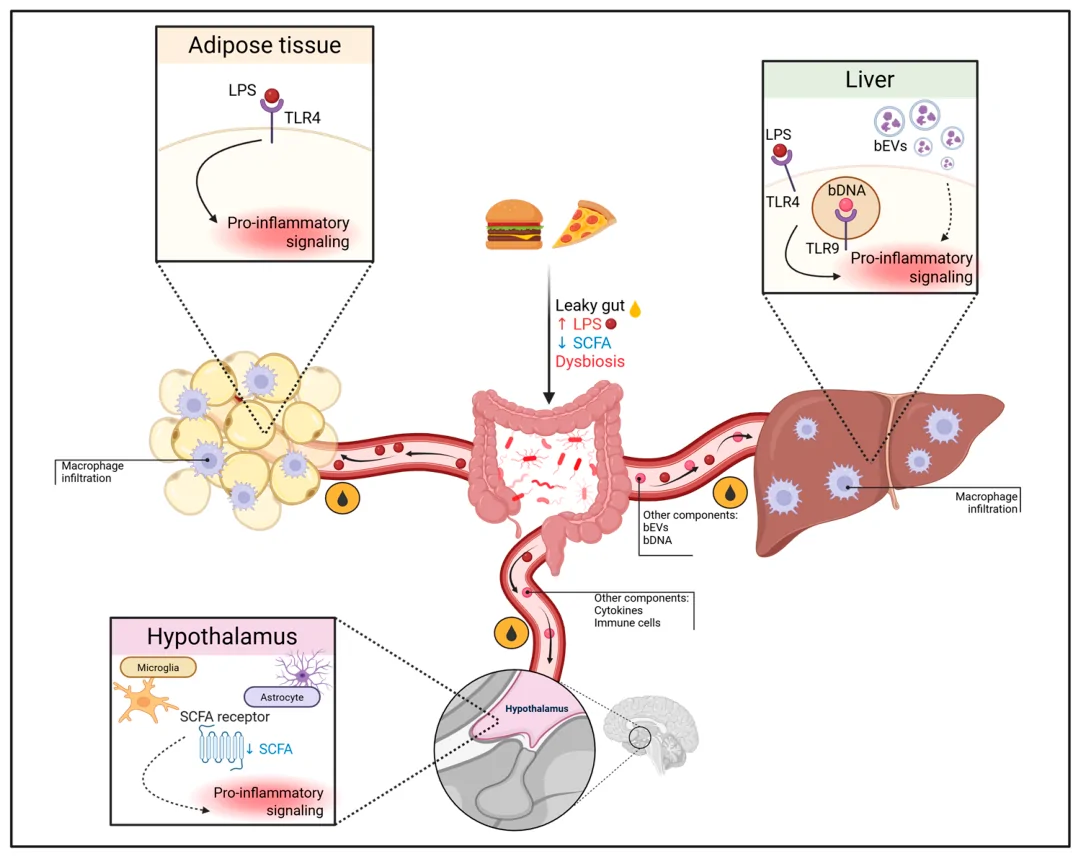
Potential pathways linking obesity-associated dysbiosis with peripheral and hypothalamic inflammation. An obesogenic diet induces gut dysbiosis, characterized by increased abundance of pro-inflammatory microbial components, including lipopolysaccharide (LPS), and altered levels of short-chain fatty acids (SCFAs). Impaired intestinal barrier integrity facilitates the translocation of microbial products, including LPS, bacterial extracellular vesicles (bEVs) and bacterial DNA (bDNA), into the circulation. These signals engage pattern-recognition receptors, including TLR4 and TLR9, and promote inflammatory responses in peripheral tissues, including adipose tissue and liver. Increased circulating microbial and inflammatory signals, together with reduced SCFA availability, may contribute to reactive gliosis and pro-inflammatory signaling in hypothalamic microglia and astrocytes. Collectively, these gut-derived signals provide a potential link between intestinal dysbiosis, systemic inflammation and hypothalamic neuroinflammation in obesity. Solid arrows indicate experimentally supported mechanistic relationships; dashed arrows indicate proposed or associative pathways for which causality remains uncertain. bDNA, bacterial DNA; bEVs, bacterial extracellular vesicles; LPS, lipopolysaccharide; SCFAs, short-chain fatty acids; TLR4, Toll-like receptor 4; TLR9, Toll-like receptor 9.

**Figure 2:**
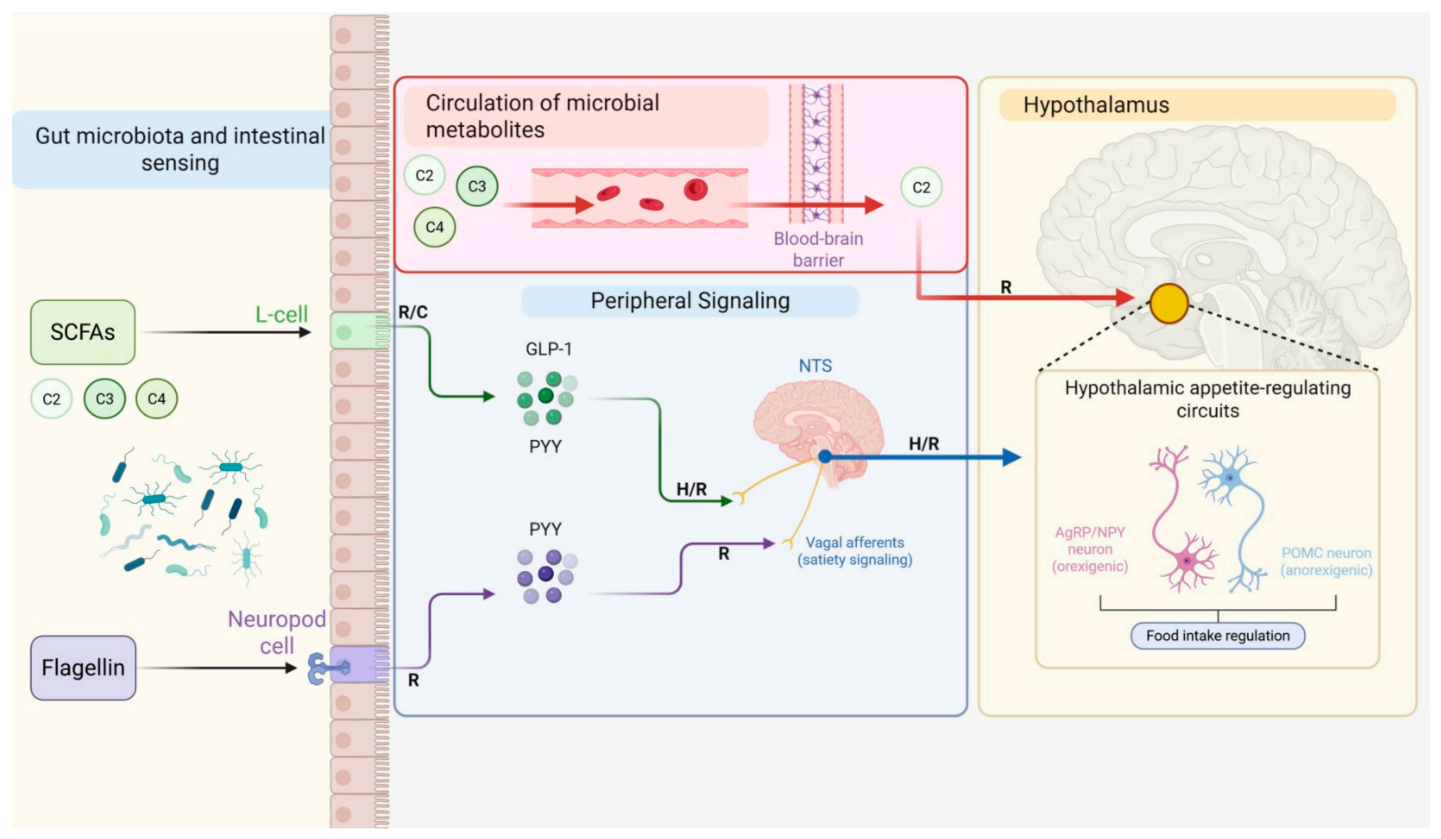
Microbiota–gut–brain signaling pathways regulating appetite. Gut microbiota-derived signals influence food intake through complementary endocrine, neural, and central pathways. In the intestine, short-chain fatty acids (SCFAs) stimulate enteroendocrine L-cells and promote the secretion of satiety-related hormones, including GLP-1 and PYY, which can activate vagal afferent pathways. Microbial flagellin can also be sensed by TLR5-expressing neuropod cells, promoting PYY-dependent vagal signaling. These gastrointestinal signals are relayed to the nucleus tractus solitarius (NTS) and subsequently integrated with hypothalamic appetite-regulating circuits. In parallel, selected microbial metabolites, particularly acetate, may reach the CNS through the circulation and blood–brain barrier, providing a potential direct route by which gut microbial metabolism may influence hypothalamic function. Together, these pathways converge on hypothalamic circuits involved in the regulation of food intake, including AgRP/NPY and POMC neurons. R, rodent studies; H, human studies; C, cellular/in vitro studies. Solid arrows indicate experimentally supported relationships represented in this scheme.

**Figure 3:**
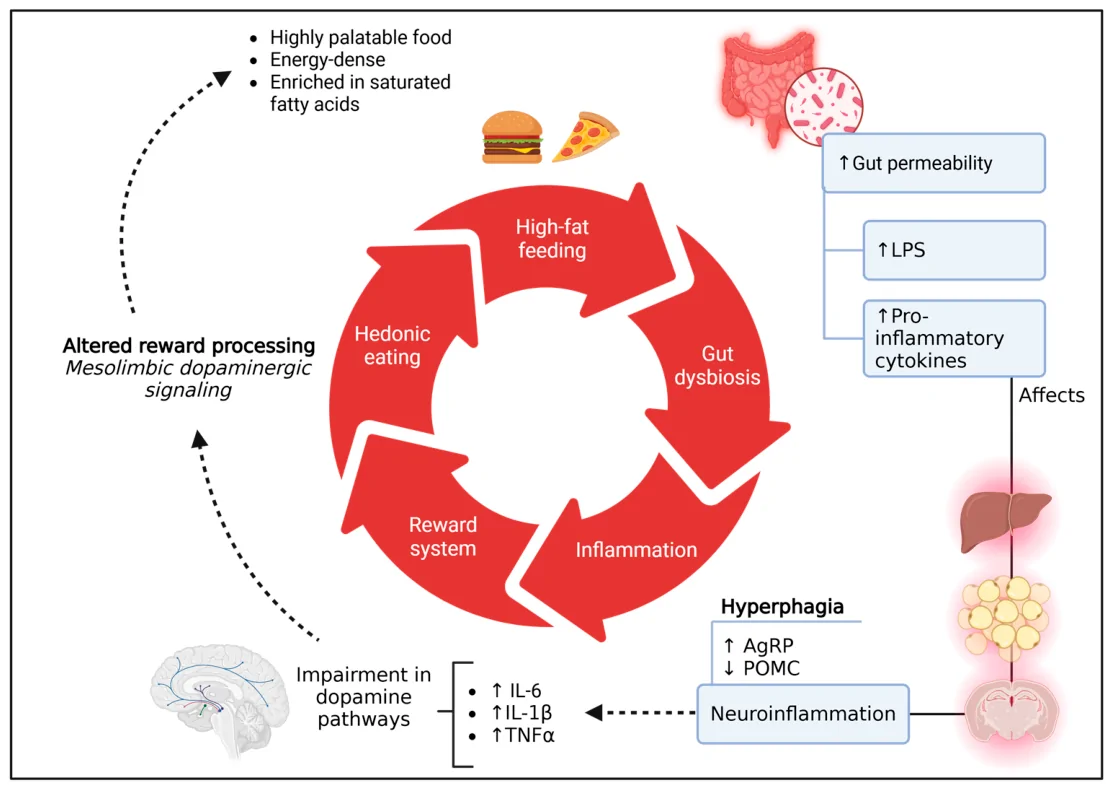
A self-reinforcing gut–brain circuit linking high-fat feeding, dysbiosis and neuroinflammation. Consumption of highly palatable, energy-dense foods enriched in saturated fatty acids promotes gut dysbiosis, which is associated with increased gut permeability, elevated circulating LPS, and enhanced production of pro-inflammatory cytokines. These alterations contribute to inflammatory responses in peripheral metabolic tissues and are associated with neuroinflammation, impaired hypothalamic appetite regulation, increased AgRP signaling, reduced POMC activity, and hyperphagia. In parallel, inflammatory and neuroimmune alterations may affect mesolimbic dopaminergic reward processing, thereby favoring hedonic eating and continued exposure to obesogenic diets. Together, these interactions define a self-reinforcing feedback framework linking gut microbiota alterations, inflammation, and dysregulated feeding behavior in obesity. Solid arrows indicate relationships supported by experimental evidence, whereas dashed arrows indicate proposed or associative pathways for which causal evidence remains limited.

## Data Availability

No new data were created or analyzed in this study. Data sharing is not applicable to this article.
